# Mixed methods research in tobacco control with youth and young adults: A methodological review of current strategies

**DOI:** 10.1371/journal.pone.0183471

**Published:** 2017-08-25

**Authors:** Craig S. Fryer, Elizabeth L. Seaman, Rachael S. Clark, Vicki L. Plano Clark

**Affiliations:** 1 Department of Behavioral and Community Health, School of Public Health, University of Maryland, College Park, Maryland, United States of America; 2 Maryland Center for Health Equity, School of Public Health, University of Maryland, College Park, Maryland, United States of America; 3 Educational Studies, School of Education, University of Cincinnati, Cincinnati, Ohio, United States of America; Hokkaido Daigaku, JAPAN

## Abstract

**Introduction:**

Tobacco use among young people is a complex and serious global dilemma that demands innovative and diverse research approaches. The purpose of this methodological review was to examine the current use of mixed methods research in tobacco control with youth and young adult populations and to develop practical recommendations for tobacco control researchers interested in this methodology.

**Methods:**

Using PubMed, we searched five peer-reviewed journals that publish tobacco control empirical literature for the use of mixed methods research to study young populations, age 12–25 years. Our team analyzed the features of each article in terms of tobacco control topic, population, youth engagement strategies, and several essential elements of mixed methods research.

**Results:**

We identified 23 mixed methods studies published by authors from five different countries reported between 2004 and 2015. These 23 articles examined various topics that included tobacco use behavior, tobacco marketing and branding, and cessation among youth and young adults. The most common mixed methods approach was variations of the concurrent design in which the qualitative and quantitative strands were administered at the same time and given equal priority. This review documented several innovative applications of mixed methods research as well as challenges in the reporting of the complex research designs.

**Conclusions:**

The use of mixed methods research in tobacco control has great potential for advancing the understanding of complex behavioral and sociocultural issues for all groups, especially youth and young adults.

## Introduction

Tobacco use among young people is a global public health issue.[1] The majority of smokers began smoking in their youth[2,3] and more than 80–90% of smokers in the United States (U.S.) began smoking in their teens.[4] Currently, approximately 50% of young men and 10% of young women smoke worldwide with annual tobacco-attributable deaths projected to rise from 5 million in 2010 to more than 10 million by 2030.[5] Tobacco-caused morbidity and mortality is the most preventable disease among humans.[6] Consequently, tobacco use among young people is a global challenge demanding concerted efforts among tobacco control experts to develop effective prevention, treatment, and cessation modalities.

To address this challenge, the field of tobacco control needs research approaches that are able to address the complex trends and contexts related to tobacco use by youth and young adults. Youth tobacco use behavior in the U.S. is consistently changing with the greater availability, marketing, and promotion of a new and diverse constellation of combustible and non-combustible tobacco products, including the emergence of flavored little cigars and cigarillos, hookah, pipes, snus, dissolvables, and e-cigarettes.[7] It is well established that the tobacco industry aggressively targets youth and young adults with multifaceted tobacco product promotions.[8,9] Additionally, how young people intentionally change dependent behaviors is not well understood within tobacco control research.[10] For instance, nicotine dependence is based on adult models and conflicting information exists on nicotine dependence as a concept among youth[11,12] despite it being a significant barrier to smoking cessation.[13] Current priority tobacco control issues with young people that need to be addressed include: (a) the development of an optimal measure of novel tobacco products (i.e., small cigar and cigarillos);[14] (b) better understanding the sensory appeal (i.e., smell and sight) of flavored tobacco products;[14] (c) the escalating trend for dual, poly-tobacco, and nonconventional tobacco product use.[15]

Mixed methods research is a methodology for collecting, analyzing, and *integrating* both quantitative and qualitative data during the research process to gain a better understanding of complex research problems.[16,17] Mixed methods approaches add multiple dimensions and rigor to more traditional single-stranded research designs because of the power of integration.[18] As a result, mixed methods research can provide stronger inferences about a finding and provides the opportunity for presenting a range of divergent viewpoints regarding the phenomena under study and engaging vulnerable populations.[17,19] Therefore, mixed methods research designs offer novel approaches to explicate the complexity of tobacco control issues (e.g., concomitant use and nicotine dependence) among vulnerable youth and young adult smokers.

There are several indicators of the need to consider mixed methods research for addressing complex tobacco control issues. The prevalence of the use of mixed methods designs has increased in recent years, particularly in the fields of the behavioral sciences[20] and health sciences[21–24] with more than 40 types of mixed methods research designs reported in the literature.[25,26] Furthermore, the Office of Behavioral and Social Sciences Research (OBSSR) of the National Institutes of Health (NIH) commissioned a leadership team in 2010 to develop a guide for investigators regarding mixed methods research. The subsequent report, *Best Practices for Mixed Methods Research in the Health Sciences*,[27] represents an important step in defining mixed methods research to the broader health sciences community. While this growth is encouraging, it is unclear how investigators are using mixed methods designs in tobacco control research to address the complex problems associated with youth and young adult populations.

Therefore, our aim was to examine and describe the use of mixed methods research in tobacco control research about youth and young adults. This methodological review contributes to understanding the adoption and use of mixed methods, which is one of five major methodological domains identified by Creswell.[26,28] Specifically, we addressed the following research questions: (a) *What mixed methods designs are investigators using to study young people in tobacco control research*?*; (b) What substantive topics are investigators addressing when using mixed methods*?*; and (c) How are investigators engaging youth in mixed methods research designs*?

There are important audiences for this methodological review. The review has the potential to help tobacco control investigators better understand key practices involved in the design and conduct of mixed methods research and the intricacies of the varied study designs. A description of current practices can also assist funding agencies in identifying mixed methods exemplars as a way to improve evaluation of this important research approach.

## Methods

Our methods build on established procedures for conducting rigorous methodological reviews and prevalence studies of the use of mixed methods research within disciplinary contexts.[29–34] These procedures guided our decisions about journal selection, search terms, inclusion criteria, and sampling.

A critical issue for methodological reviews is the identification of the appropriate journals for the search. While some methodological reviews have examined only one journal[35], it is most common to assess several journals.[34] Additionally, authors often focus on the leading journals within specific disciplines for the review in order to identify high-quality research applications.[34] Building upon highly-cited reviews in other fields,[23,29,36] we decided to limit our search to specific journals that publish tobacco control research to focus the scope of our review and ensure results were replicable and face validity was high. Our aim was to review the best subset of articles from which to learn; exemplars in the field. We solicited the opinions of six nationally-known tobacco research experts to determine the best journals in the field of tobacco control with interest in young populations. Combining this consultation with our expertise and years of experience working in the field of tobacco control research, five high quality, peer-reviewed journals were identified to be included for analysis: Addictive Behaviors, Health Education Research, Nicotine and Tobacco Research, Social Science and Medicine, and Tobacco Control. To ensure that we were not missing a significant source of mixed methods articles, we conducted informal searches of other high-quality, peer-reviewed journals (i.e., Addiction and Preventive Medicine), but did not locate articles that met all study inclusion criteria.

### Inclusion criteria

The inclusion criteria of our methodological review encompassed three main designations: youth and young adults, tobacco control research, and mixed methods research defined as:

1. Youth or Young Adults—Articles involving youth between the ages of 12–17 years and/or young adults aged 18–25 years.[6]
2. Tobacco Control–Articles involving empirical research regarding any aspect of tobacco use and nicotine dependence.
3. Mixed Methods Research–Articles reporting the use of research designs that collect, analyze, and integrate quantitative and qualitative data within a single study or multiple phases of a program of research[16].

### Sampling

**Fig 1:**
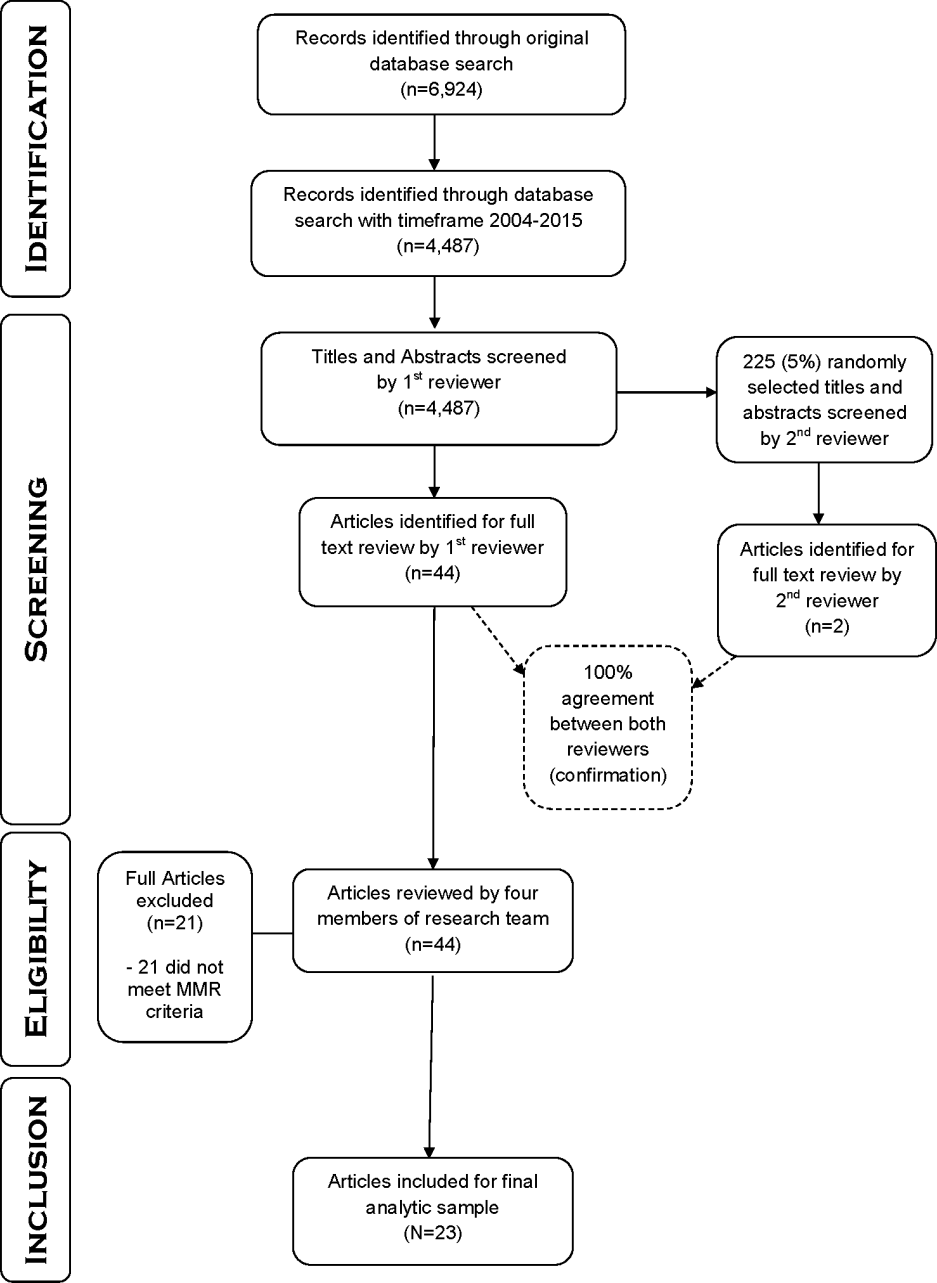
Four-step article search and screening process flowchart.

The sampling phase utilized a four-step process: identification, screening, eligibility, and inclusion. An illustration of this process is presented in Fig 1.

The first step was sample identification. The scientific research articles were identified through a broad, multifaceted search strategy. On October 1, 2016 we implemented our search of tobacco control empirical studies that used mixed methods research designs with young people published during January 1, 2004 to December 31, 2015. We searched the selected five journals for articles that matched our first two inclusion criteria (participant age and tobacco control) using PubMed. We chose PubMed as our database because of its comprehensiveness (5,153 journals in *Index Medicus* and 479 non-*Index Medicus* journals) and management by the National Institutes of Health’s U.S. National Library of Medicine. This search used broad subject terms, but did not include the term “mixed methods research” because mixed methods experts have cautioned against relying on this term when searching electronic databases as it may result in missing publications that combine quantitative and qualitative methods without self-identifying as mixed methods [34]. The search strategy used was: (((((((((youth or Adolescen* or "young adult")) AND ("Nicotine & tobacco research: official journal of the Society for Research on Nicotine and Tobacco"[Journal])) OR "Tobacco control"[Journal]) OR "Health education research"[Journal]) OR "Social science & medicine (1982)"[Journal]) OR "Addictive behaviors"[Journal])) AND (Tobacco OR cigarette* or Smok*[Title]). This search of titles yielded 6,924 records. Next, we applied our year range filter (2004–2015) and the results were reduced to 4,487 records. The full yield of 4,487 records was then assessed against our three inclusion criteria.

The second step was screening. Abstracts were obtained for each of the 4,487 titles. The second author (ELS) screened the entire sample by reviewing abstracts to identify all articles that potentially met the inclusion criteria for full review. Often the second and first author (CSF) met to discuss screening decisions. Letters to the editor, systematic reviews, commentaries, special communications, research briefs, and editorials were excluded. To confirm the accuracy of the screening, a randomly selected subsample of 5% (225 titles and abstracts) of the original 4,487 results was sent to the third author (RSC). RSC used the identical inclusion criteria and identified two abstracts among the 225 that warranted a full review. The two abstracts matched those identified by ELS’s review, resulting in a 100% agreement for the 225 abstracts. The screening yielded 44 abstracts of articles eligible for full review.

The third step was to determine the eligibility of all 44 abstracts by obtaining their respective full-text copies. The full articles were then examined and discussed by all four members of the research team. From this review, we excluded 21 articles that reported only qualitative or quantitative methods and thus, did not meet our inclusion criteria for mixed methods research.

The fourth step assessed the remaining articles for inclusion in the sample. Our process resulted in a final sample of 23 articles for the methodological review.

### Data coding

The first author (CSF) in consultation with the senior author (VPC) developed a codebook to examine the articles based on categories used in methodological reviews of the use of mixed methods in other topical areas.[29,36,37] The codebook was comprised of names and brief descriptions highlighting seven different article features: Substantive Content, Quantitative Component, Qualitative Component, Mixed Methods Features, Youth Engagement Features, Other Issues, and Reflections. Details of the codebook, including the dataset are provided in S1 File.

Two team members (ELS and RSC) were responsible for the primary coding of all articles. Each article was given a randomly generated identification number and randomly assigned to ELS or RSC. They independently coded each assigned article using an Excel spreadsheet accessible by all team members through GoogleDocs^TM^ including direct quotes from the articles and the coder’s interpretations of the information. After initial coding of each article was complete, the senior members (CSF AND VPC) were randomly assigned to each article, reviewed and recorded information and discussed any discrepancies with the primary coder. Final consensus was reached among the full team through discussion for each article in the dataset.

### Data analysis

To characterize the current state of the use of mixed methods research with youth and young adults in tobacco control research, the studies were examined to assess research methods and designs, research participants, sampling, approaches to analysis, and the tobacco control topic or behavior investigated. Each team member was assigned a section of the data codebook to analyze and provided a summary of their findings. During weekly meetings, each team member presented their findings and the remaining three members provided critical feedback. The result was an inductive analysis process that examined how specific features of each article holistically described the mixed methods research being conducted in tobacco control with young people.

## Results

Table 1 lists the 23 articles included in this methodological review. The articles were published between 2004 and 2015. The majority (n = 16) were published between 2006 and 2011 and by scientists from the U.S.A. (n = 13) and the United Kingdom (n = 7). We also identified mixed methods studies with young people conducted in India (n = 1), Israel (n = 1), and New Zealand (n = 1). The country affiliation of the authors of these studies may have differed from the location of the research such as Nichter.[38] The number of co-authors ranged from two to nine. The preponderance of the first authors were in the behavioral sciences (n = 14) including anthropology, psychology, and public health. Others were from the fields of medicine (n = 6), marketing (n = 2), and statistics (n = 1).

**Table 1:** Tobacco control characteristics of articles reviewed (N = 23).

Lead Author (Year of Publication)	Country Affiliation of First Author	Topic	Population	Recruitment and Engagement Strategies
Acosta[39] (2008)	USA	Early cigarette use experiences among college students	Youth (17) and Young Adults (18) entering college as freshmen of mixed gender	**Quan**: Passive (Screener given to all incoming freshmen via email)(n = 163)
				**Qual**: NEI^a^ (n = 26)
Amos[40] (2006)	UK	Experiences with and attitudes towards addiction and cessation	Youth 16–17 and Young Adults 18–19 of mixed gender	**Quan**: Active (Adults at various community sites utilized to recruit; researchers also engaged participants directly) (n = 99)
				**Qual**: Active (see above) (n = 99)
Audrey[41] (2006)	UK	Adolescent perspectives of an intervention to reduce smoking	Youth in Year 8 (approximate ages 13–15) of mixed gender who were followed for 2 years post intervention since adolescence	**Quan:** Passive (all students in Year 8 of schools) (n = 10,370)
				**Qual**: Active (Peer nominations to recruit) (n = 978)
				**PA**^c^: peer nomination and counseling
Berg[42] (2010)	USA	Definitions of what is a "smoker”	Young adults 18–25 who were current smokers of mixed gender	**Quan**: Passive (recruited through email) (prior quan study n = 2,700)
				**Qual**: Both Active and Passive (they invited students via phone & email) (n = 73)
Elsey[43] (2015)	UK	Social norm approaches for preventing cigarette smoking in schools	Youth 12–13 of mixed gender	**Quan:** Passive (recruited through schools) (n = 595)
				**Qual:** Active (approaching students who helped design the campaign or experienced the campaign) (n = 96)
				**PA**^c^: collaboration with local arts organization
Gendall[44] (2011)	New Zealand	Interpretations of Tobacco brands	Young adults 18–24 of mixed gender	**Qual**: both Active (Direct approaches through kinship networks) and Passive (Posters) (n = 66)
				**Quan**: both (see above) (n = 66)
Goenka[45] (2010)	India	Evaluation of a tobacco prevention program	Youth of approximate ages 11–15 (from grades 6–9) of mixed gender	**Quan:** (randomized through schools) (n = 5,564)
				**Qual:** (n = NEI^a^)
				**CSA**^d^: mascots and skits
Kong[46] (2015)	USA	Understanding reasons for trying and quitting e-cigarette use	Youth in middle schools, high schools, and college students of mixed gender	**Quan:** Passive (recruited through schools) (n = 5,045)
				**Qual:** Passive (flyers handed out at lunch time (MS, HS, and random college classes) (n = 127)
Lee[47] (2010)	USA	Smoking of cigarettes, cigars and blunts	Youth 15–17 and Young Adults 18–24 of mixed gender	**Quan**: Active (agency referrals and snowball sampling) (n = 164)
				**Qual**: Active (see above) (n = 164)
Lee[48] (2011)	USA	Adolescent characterizations of smoker types	Youth 12–16 of mixed gender	**Quan**: Passive (recruited through schools) (n = 372)
				**Qual**: Passive (selected students from the survey) (n = 40)
Levin-Zamir[49] (2011)	Israel	Media Health Literacy (MHL) and smoking	Youth approximate ages 13, 15 and 17 year olds (from grades 7, 9 and 11) of mixed gender	**Quan** (n = 1,316) **and Qual** (n = 60): Passive (Parents gave passive consent)
Levy[50] (2010)	USA	Smoking communication between parents and their depressed adolescent children	Youth 14–17 and Young Adults 18–19 of mixed gender; current smokers with depression	**Quan**: both Active (office staff) and Passive (flyers at primary care centers and local health centers)(n = 15 parent/child dyads)
				**Qual**: both (see above) (n = 15 parent/child dyads)
MacPherson [51] (2006)	USA	Adolescent definitions of change in smoking behavior	Youth 14–17 and Young Adult 18 year-old smokers of mixed gender	NEI^a^ (Recruited through high schools)(n = 94 for both phases)
Mair[52] (2006)	UK	Reliability of young peoples’ accounts of smoking	Longitudinal data of youth 13–15 of mixed gender who were followed for 10 years since childhood	**Quan:** Passive (survey given to all students in schools) (n = 208);
				**Qual:** Active (Recruited by teachers) (n = 208)
Molyneux[53] (2005)	UK	Designing Cessation Programs for young smokers	Youth 13–16 of mixed gender who were current smokers who desired to quit	**Quan**: NEI^a^ (Recruited through secondary schools) (n = 4,065)
				**Qual**: Passive (Parents provided passive consent and students meeting behavior criteria were sent letters) (n = 135)
Moodie[54] (2011)	UK	Perception of cigarette packaging	Young adults 18–24 and older 25–35 smokers of mixed gender	**Quan**: Active (Door-knock method in selected postcodes) (n = 48)
				**Qual**: Active (phone calls) (n = 18)
Nasim[55] (2014)	USA	Videos of cigar product modification	Videos and comments posted online	N/A^b^ (video content review) (n = 26 videos and n = 2,457 comments)
Nichter[38] (2004)	USA	Perceptions of tobacco products and patterns of use	Youth 16–17 and Young Adults 18–23 of mixed gender (although the majority of participants were male)	**Quan**: Active (Recruited through schools) (n = 1,587)
				**Qual**: Active (key informant interviews, focus groups, and observations); (n = 25 interviews; NEI^a^ for focus groups)
O'Neill[56] (2013)	USA	Motivations for genetic testing for lung cancer risk	Youth 17 and Young Adult 18–22 smokers of mixed gender	**Quan**: Mix of both Active (Approaching people on campus) and Passive (Advertisements) (n = 128)
				**Qual**: Both (see above) (n = 128)
Primack[57] (2009)	USA	Impressions of antismoking media literacy education	Youth approximate ages 14 & 15 of mixed gender	NEI^a^ (Recruited through high schools) (n = 531 for both phases)
Sorensen[58] (2004)	USA	Development of a worksite smoking intervention	Youth 15–17 and Young Adults 18 of mixed gender who worked at least 5 hours a week	**Quan**: Active (approached by store contacts and research staff) (n = 375)
				**Qual**: NEI^a^ (n = 41)
				**PA**^c^: teen advisory boards
				**CSA**^d^: workplace setting
Tiffany[59] (2007)	USA	Smoking trajectories among college freshmen	Youth (17) and Young Adults (18) of mixed gender entering college as freshmen	**Quan**: Passive (recruited using postcards) (n = 912)
				**Qual**: Passive (selected from survey results) (n = 16 interviews; NEI^a^ for focus groups)
Turner[60] (2006)	UK	Peer influence on adolescent smoking behaviors	Youth 13 and 15 years of mixed gender	**Quan**: Passive (Opt-out note sent home to parents) (n = 896)
				**Qual:** Active (participants to bring friends from class) (n = 136)

^a^ NEI: Not Enough Information

^b^ N/A: Not Applicable

^c^ PA: Participatory Approach

^d^ CSA: Culturally-Sensitive Approach

Next, we describe the substantive content, youth engagement methods including recruitment, and mixed methods research design of these 23 articles.

### Substantive content

Of the 23 articles reviewed, two articles reported on setting-specific smoking prevention programs[45,58] while five reported on perceptions of smokers and various tobacco products.[42,43,48,50,51] Five articles focused on smoking prevalence and behavior[39,46,47,59,60] while three explored the influences of tobacco marketing and branding.[38,44,54] Three articles [40,41,53] examined aspects of smoking cessation including services, two examined the use of media literacy to prevent smoking [49,57], and the remaining articles focused on cigar product modification,[55] motives for and against genetic testing[56] and the reliability and validity of self-reported data.[52] Furthermore, two-thirds (n = 14) of the articles reported on the use of cigarette smoking only and one reported on e-cigarette use and cessation only among middle school, high school, and college students.[46] The authors of five articles focused on the use of cigarettes and another tobacco product such as smokeless tobacco, cigars, hookah, bidis, and cloves. One article reported on the use of cigarettes and cannabis[47] and another on cigar modification.[55]

### Youth engagement through recruitment and intervention

The populations of young people represented in the 23 articles were varied and reflected the international scope of tobacco research. Given that many countries worldwide prohibit sales and restrict access to youth for tobacco products[38] as well as the increasing denormalization of the social acceptability of smoking, engaging young people in tobacco control research is challenging.[38,61] Therefore, we examined how the investigators engaged their participants through the phases of planning, recruitment, and retention in the mixed methods research.

We were particularly interested in recruitment strategies used in these studies. The investigators utilized both passive (e.g., flyers, email, posters) and active recruitment methods (e.g., staff of the schools or community organizations, referrals, presentations to groups) to engage young people in their research studies (Table 1). A little more than two-thirds (n = 16) utilized the school setting (i.e., middle or high school and college) for recruitment. The study samples reflected the ethnic distribution of the geographic locations, yet few studies had ample numbers of young people across racial and ethnic categories to perform group comparison analyses. The majority of the research teams used traditional recruitment methods (e.g., flyers and email) and the recruitment strategies supported the diverse research designs (cross sectional or longitudinal), sampling techniques (convenient or purposeful), data collection methods (quantitative or qualitative), and populations of interests (youth or young adults).

Furthermore, several of the articles engaged youth through the development, implementation, or evaluation of tobacco control interventions. [41,43,45,58] In this review, investigators were strategic in how they engaged youth in their research employing participatory and culturally-sensitive approaches. The participatory approaches varied in the studies. For example, Audrey et al [41] employed a peer nomination process and counseling techniques to identify and gain peer support for a student-led smoking prevention program. The most nominated students were trained as peer supporters and charged to recruit a wide range of friendship groups within a ten week period. The team reported 87% of those invited accepted the position, were trained, and completed their duties and 86% attended all follow-up sessions. Elsey and her research team [43] examined the feasibility and acceptability of using a social norms approach in five schools among seventh graders to prevent smoking. Findings demonstrated youth had significant misperceptions between self-reported and perceived smoking among their peers in the same grade. As a result, they collaborated with a local arts organization to both engage and assist students in the development of their own social norm campaigns to correct misperceptions of peer smoking. Investigators in this review also used culturally-sensitive approaches to engage young people in their research. For example, Goenka and colleagues[45] conducted a process evaluation of a classroom-based, tobacco prevention intervention in India. The intervention addressed both smokeless and smoked forms of tobacco among sixth to ninth graders and was tailored to Indian culture in context, content, and communication. The seven-session curriculum utilized mascots (*Disha* and *Deepak*), posters, and the creation of skits to sustain the interests of youth. Results indicated higher levels of student and peer leader communication and higher levels of student participation in discussion. Finally, one study used a combined participatory and culturally-sensitive approach. Sorensen et al[58] investigated the use of a workplace intervention to increase knowledge about the harms of smoking among teenage smokers. The intervention developed teen advisory boards to facilitate discussions about the negative impact of smoking on health with study participants. In addition, participants were recruited from and the study was implemented in 10 grocery stores in Boston, USA. As such, the culture of the workplace setting was an important component to the study design. Contrary to the team's predictions, most teen workers preferred to quit on their own, suggesting interventions with working teens may not be maximally effective.

### Use of mixed methods research

Our analysis of the articles focused on the researcher’s reports of their use of mixed methods research. Consistent with our definition of mixed methods research, we examined the quantitative and qualitative components of each study and how the authors integrated these two components. Based on this information, we classified each study in terms of one of the five major mixed methods research designs discussed by Creswell and Plano Clark.[16] This information is summarized in Table 2 and discussed in the sections that follow.

#### Quantitative components

Each of the reviewed mixed methods studies included a quantitative component that reflected the researchers’ decisions about sampling, collection, and data analysis. A range of sampling strategies were used for the quantitative component of the studies including purposive sampling,[40,44,46,60] probability or random stratified sampling,[49,54] multi-stage sampling,[55] and a combination of sampling methods.[47] We concluded that the remaining 15 studies utilized convenience sampling based on the described contextual information. The sample sizes of the quantitative strands of the studies varied greatly. The smallest sample included 15 parent/child dyads[50] and the largest study enrolled 10,370 participants.[41]

**Table 2:** Overview of mixed methods design features.

Mixed Methods Design^a^	Reviewed Studies	Rationale and Value	Timing	Priority Options	Mixing Strategies
**Concurrent** **(Quan + Qual)**	[39,40,44,47,48,51,52,55–57,59]	To generate generalizable results combined with in-depth explorations for cross-validation, confirmation, or completeness	Concurrent: quantitative and qualitative strands administered at same time	Equal, quantitative, or qualitative	Merging results during data analysis and/or interpretation
**Explanatory Sequential** **(Quan→Qual)**	[42,43,45,50,53,54]	To explain the mechanisms behind quantitative results with qualitative findings or build on quantitative trends with qualitative examination	Sequential: quantitative strand administered first and followed by qualitative strand	Quantitative or qualitative	Connecting from quantitative results to qualitative data collection and connecting results during interpretation
**Exploratory Sequential** **(Qual→Quan)**	[46,49,58]	To explore a phenomenon before measuring with quantitative methods to verify or build on qualitative findings with quantitative results	Sequential: qualitative strand administered first and followed by quantitative strand	Qualitative or quantitative	Connecting from qualitative findings to quantitative data collection and connecting results during interpretation
**Embedded** **QUAN(qual)**	[41]	To examine the intervention process within an experimental test of intervention outcomes	Concurrent	Quantitative	Embedding qualitative data within an experimental design
**Multiphase** **(Quan→qual) →[qual+quan]**	[38,60]	Multiple phases needed to thoroughly address study objective(s)	Combination of sequential and concurrent	Varies by strand	Combination of merging and connecting

^a^ The mixed methods research designs highlighted in the first column of Table 2 are derived from Creswell and Plano Clark.[16]

The majority of the studies utilized a survey or a questionnaire for the quantitative data collection. Some studies combined a survey or questionnaire with another quantitative component such as a video activity,[49] ranking tobacco packaging,[54] and a salivary cotinine sample.[41] Two studies used “web assessments” to collect data at more than one time point.[39,59] Three studies utilized validated measures such as the Beck Depression Inventory and the Teen Smoking Questionnaire.[50,51,53] Several studies used more innovative ways to collect quantitative data, such as quantifying participants’ brand attribute associations to create a perceptual map.[44]

#### Qualitative components

Each of the reviewed mixed methods studies also included a qualitative component that reflected the researchers’ decisions about sampling, collection, and data analysis. In terms of sampling, 13 studies used a subset of the quantitative sample for their qualitative data collection (e.g.,[41,48,53]). The subsets were chosen from the full sample size in different ways. One study relied on participants to volunteer for the qualitative portion and bring a friend,[60] whereas two studies used nominations to select which students to include in the qualitative phase of the study.[41,49] Nine studies used the same sample in the qualitative and quantitative components (e.g., [40,44,57]). The sample sizes for the qualitative components ranged from 18 to 531 participants.

Several studies combined qualitative methods to examine both the opinions of the group and individuals, such as focus groups and interviews.[53] Two studies utilized more than two qualitative methods, such as combining interviews, focus groups, informal discussions and ethnographic fieldwork.[38] Some studies employed uncommon combinations–for instance focus groups with media diaries[49] or semi-structured interviews with adjective cards to describe participants’ smoking experiences.[39] Many of the studies provided little information about the specific qualitative data analysis. Six studies directly mentioned performing a thematic analysis, all with varying levels of detail.[38,40,44,55,56,60] One study team cited a focus group guide[62] to describe their analysis[42], while another performed a descriptive content analysis on YouTube videos and a thematic analysis on the comments associated with each video.[55]

#### Reasons for and value of mixing methods

Central to the use of mixed methods is integrating the quantitative and qualitative components of a mixed methods study. This integration often starts by the researchers specifying a rationale for mixing methods. Due to the complexity that mixed methods brings to research, it is important for researchers to describe the rationale and value that the mixed methods approach brings to a study.[63,64] Of the 23 articles reviewed, only three explicitly stated the authors’ reason for using mixed methods.[41,49,57] Therefore, we analyzed the remaining articles to uncover the implicit rationale for each study.

Common rationales included triangulation (n = 7) and complementarity (n = 7). Triangulation involves corroborating results from the quantitative and qualitative methods to obtain more validated conclusions.[63] One study[47] used mixed methods to triangulate patterns of behavior by comparing quantitative and qualitative data to identify and understand “culturally relevant meanings” that shaped participants’ substance use behaviors. Complementarity is used when quantitative and qualitative methods examine different aspects of the same phenomenon resulting in a deeper, more complex understanding of the topic.[64] For instance, investigators[56] discovered novel information from open-ended items on a questionnaire that explained differences in quantitative and qualitative findings regarding interest in genetic testing to determine cancer risk. Some studies used the results of one method to develop or inform the use of subsequent methods.[64] Examples of development include needing quantitative results to select participants[53] or using qualitative results to develop a new measure.[49]

#### Timing of components

Timing refers to the order in which the qualitative and quantitative components were used in a mixed methods study.[16,65] Some of the articles did not include a clear description of when the strands were administered, which made timing challenging to determine. Based upon our interpretation, many studies used concurrent timing (n = 12), which occurs when researchers administer the qualitative and quantitative components concurrently. For example, researchers[44] collected quantitative (questionnaire) and qualitative (focus groups) data within the same session.

Nine studies used sequential timing, which occurs when researchers fully implement one phase (quantitative or qualitative) before the other so the second phase depends on the results of the first phase. For example, in a study to characterize and measure media health literacy,[49] the researchers first implemented the qualitative component and then used those results to create a questionnaire utilized in the subsequent quantitative phase. Two studies used multiphase timing by combining multiple concurrent and/or sequential components.

#### Priority

Priority describes the relative importance of the quantitative and qualitative components for addressing the overall study purpose.[16] In a mixed methods study, the two components may have equal weight in addressing the study purpose or one method may be given more weight than the other. We assessed studies as having quantitative priority (n = 11), qualitative priority (n = 7), and equal priority (n = 5). For example, Nichter et al[38] assigned priority to the quantitative methods in their study of perceptions of tobacco use and behavior associated with use because the article contained many tables of quantitative results, but no qualitative data illustrations. Audrey and colleagues[41] demonstrated qualitative priority because they used extensive analysis of peer leader diaries and focus group data to conduct a process evaluation of a peer support and intervention program. Equal priority was illustrated by a study of self-report measures of youth smoking in which researchers used data from questionnaires and semi-structured interviews to examine inconsistencies in reliability between measures.[52]

#### Mixing strategies

Mixing strategies can be classified into three broad categories: merging, connecting, and embedding.[16] Merging occurs when the qualitative and quantitative results are combined during analysis. Connecting occurs when the results or findings from one strand are used to design the data collection of another strand. Embedding occurs when a secondary qualitative or quantitative component is added to an overall quantitative or qualitative design. For example, researchers may embed a qualitative component into an experiment or a quantitative component into a case study.

Eleven studies used merging to integrate results. For example, researchers[40] described the merging of the quantitative and qualitative data sets as, “Responses to questions on quitting from the structured questionnaire were analyzed in SPSS and contrasted with what was said in the interviews” (p.183). Another form of merging occurs when one type of data is transformed into another in order to facilitate analysis and comparison. Primack, Fine, and Yang[57] illustrated this type of mixing, as the researchers collected qualitative data and transformed the qualitative results into quantitative data in order to “illuminate results of the outcome evaluation” (p. 326).

Connecting was used as a mixing strategy in nine articles. For example, one team[53] selected participants for the qualitative strand based on results from the quantitative strand. The authors described the desire to interview individuals of “both genders who wanted to give up smoking and were in three older year groups and from a range of different schools” (p. 543). Once the quantitative results were collected, the researchers purposefully selected six representative schools and all students were invited to participate in the qualitative phase of the study.

Investigators used embedding in a study of a peer-led smoking intervention.[41] The qualitative data collection occurred within the context of an experiment and included diary entries, semi-structured interviews, and focus groups that were analyzed and used to augment the quantitative data and complete the process evaluation. Two studies used a combination of mixing strategies.[38,60]

#### Overall mixed methods design

As indicated in Table 2, we applied the mixed methods design typology of Creswell and Plano Clark[16] and identified examples of five different mixed methods designs: concurrent, explanatory sequential, exploratory sequential, embedded, and multiphase.[16] Of note, no study reported the use of the transformative mixed methods design discussed in Creswell and Plano Clark.[16] Eleven studies used a variation of the concurrent design. The concurrent design (Quan + Qual) typically entails administering the quantitative and qualitative strands concurrently, emphasizing each strand equally, and merging the results from both strands during the final analysis and interpretation for the purposes of triangulation and complementarity.[16] In a study on self-reported smoking data, survey and focus group results were given equal weight and used for comparison to test the reliability over time and across settings.[52]

Six studies used the explanatory sequential design (Quan→Qual). This design entails collecting and analyzing quantitative and qualitative data sequentially where the qualitative phase builds on the quantitative results and is used to help explain the quantitative results.[16] In a study of perceptions of cigarette packaging,[54] the investigators used an explanatory sequential design to first measure the impact of non-branded cigarette packs and then explain why the impact occurred by providing smokers the opportunity to explain and expand upon their quantitative results.

As described in Creswell and Plano Clark[16], the remaining six studies used the exploratory sequential approach (Qual→Quan),[46,49,58] the embedded QUAN(qual) design,[41] or the multiphase design (Quan→qual) →[qual+quan].[38,60]

#### Mixed methods reporting issues

The main issues with reporting were: i) the use of clear language and ii) the scope of the information reported. In many of the articles, the authors did not clearly distinguish the different study components of their work. Some authors made explicit choices about their language use, which facilitated understanding the study’s components. For example, one research team[60] explicitly discussed their choice of terminology to distinguish study participants based on their participation in the quantitative or qualitative study strand. They wrote, "In this paper, in order to clarify whether we are referring to individuals who were surveyed or those who were interviewed, the former will be referred to as *pupils*, the latter as *participants*" (p. 1517, italics in original).[60] The other reporting issue was the scope of information that was included in the articles. Many studies were extensive and reported only a fraction of the studies’ information in the articles. Several strategies were noted to deal with the issue of scope in these situations. Some authors described the methods of the full study but then reported only a subset of the study results, such as results for one phase[42,54] or one wave of data collection.[57] Other authors specifically referred to information available outside of the article to supplement the description of the study’s methods, such as reported in other publications,[48] available online,[45] or to contact the corresponding author.[44]

## Discussion

Population-based systematic reviews examining access to tobacco,[66] smoking initiation,[67] tobacco control interventions and social inequalities,[68,69] longitudinal studies and smoking cessation trials,[70,71] and tobacco control policies[72] have been published for more than a decade. To our knowledge, this is the first methodological review of the use of mixed methods research designs in tobacco control being employed with youth and young adults–which we consider to be novel and an important contribution to the scientific literature. Our team identified 4,487 articles in five journals and synthesized evidence from 23 published articles representing 22 mixed methods studies in this review. The total yield is less than 1% of the original sample amplifying that mixed methods research represents a small, yet important body of work within tobacco control research with youth and young adults.

In summary, studies combining qualitative and quantitative methods were successfully being implemented with young people in tobacco control research both globally and across many disciplines (see [45,47,51,54]). In the field, investigators addressed relevant issues facing young people across the continuum of prevention, treatment, and cessation utilizing multiple variations of five of the six major mixed methods research designs.[16]

Of the 23 studies in this methodological review, the most common approach was some variation of the concurrent design. These findings are congruent with other methodological reviews that report the increased implementation of mixed methods designed studies across many disciplines[22,23,73,74] and the concurrent design identified as the most commonly utilized mixed methods design.[29,75] Additionally, although most of the investigators utilized traditional data collection procedures (i.e., surveys, focus groups, and individual interviews), a smaller group of the articles described other combinations of data collection methods, including video activities, biological sample collection, and perceptual mapping. Plano Clark [73] found similar results in a review of federally funded, health-related grant applications, yet noted that this was not the case in a review completed two years before in family sciences.[29] This suggests that as mixed methods research designs increase in popularity and use, the more intricate features of the designs may become.

As previously mentioned, mixed methods research can provide stronger inferences about a finding and offer insights regarding the phenomena under study that could not be gleaned from the quantitative or qualitative component alone.[16,17,25] This review uncovered several exemplars of such inferences for tobacco control. For example, Turner and colleagues[60] utilized a multiphase mixed methods research design in their examination of peer group influence on school smoking rates. While their qualitative findings complemented those of the quantitative analysis regarding attitudes of smoking among non-smoking girls, the mixed methods analysis provided a deeper understanding of the mechanisms operating among these groups of girls at the two socioeconomically different schools. Specifically, the processes of selection (i.e., modeling) were operating at one school, while those of influence (i.e., coercion) were found at the other school. A different, but equally salient example of the power of mixed methods study designs was found in the work of Mair et al.[52] This investigative team utilized a concurrent mixed methods research design with longitudinal qualitative and quantitative data to assess the reliability of self-reported smoking data among young people. Once they merged their study results during data analysis and interpretation, they learned that the ‘inconsistencies’ found statistically were actually important self-characterizations young people had of themselves overtime. As a result, they concluded that the ability to identify inconsistencies was a strength of the research. More importantly, the inconsistencies were not related to the self-reported data, but investigators’ tendencies to employ analytical models that are not germane to the lived experience of the young participants.

Although a few investigators reported creative ways to engage young people in their research–utilizing the arts, peer counseling, and culturally-appropriate mascots, [41,43,45],overall our sample lacked innovative exemplars of youth engagement. In an effort to protect the health of youth and young adults, tobacco control investigators must be as effective in engaging youth in research as the tobacco industry has been in attracting young people to the multitude of new tobacco products continuously emerging on the global market. Equally important are contextually appropriate and sensitive methods to examine the feasibility, process, and outcomes of such interventions. Our sample provides examples of using mixed methods research designs to do that [43,54,57] and these techniques should be applied to develop more innovative intervention approaches such as the use of new media strategies like social media platforms (i.e., Instagram or Youtube) or smartphone applications (i.e., Smokerface or QuitStart). Furthermore, the use of existing anti-smoking programs like the truth ® #FinishIt campaign and the Fresh Empire ® music contests and events to name a few, can be novel mechanisms in tobacco control intervention designs to engage young adult interests across the continuum of planning, recruitment, and retention.

While we applaud the successful use and variety of mixed methods designed studies in this review, we found unevenness and inconsistency in the reporting of this important work in the published tobacco control empirical literature. We reflect upon three broad, pertinent issues found in our review: lack of common terminology, lack of information, and the lack of clear descriptions and explanations of procedures that impact our ability to assess the quality and rigor of these published works.[22]

### Lack of common terminology

Since the early 1990s, the field of mixed methods research has developed a wealth of resources regarding the conceptualization, design, and implementation of mixed methods studies.[16,25,73,76–78] Despite such resources, the researchers of the studies examined herein often did not use common terminology in the reporting of their work.[22] Case in point, unlike methodological reviews in other disciplines,[73,74] very few of the authors used mixed methods terminology such as ‘mixed methods’ or self-identified their study as ‘quantitative and qualitative’ in the description of their research. As reported in the Methods section of this article, because there was no consistent language to identify mixed methods studies, we decided to implement a broad, multifaceted search strategy in a stepwise procedure. Thus, we used simple key words in our search and then assessed a larger sample of articles to find information-rich examples of mixed methods studies. The lack of clear terminology hampers the proliferation of innovative techniques and prohibits learning. By understanding the many possible mixed methods approaches, including advantages and challenges, tobacco control investigators will be able to better choose and articulate their mixed methods designs. It is imperative that investigators use common terminology so that the work can be identified, assessed, and ultimately replicated in the pursuit of scientific advancement. We recommend that authors using mixed methods approaches name their approach “mixed methods research” and that they provide a formal mixed methods design name (e.g., explanatory sequential mixed methods design) as discussed in the literature and used in this review.[16]

### Lack of information

Another overarching concern was the lack of information provided to effectively ascertain the study implementation procedures. For example, some authors mentioned the setting in which recruitment took place (i.e., a school or a workplace), yet did not describe the *process* by which young participants were recruited to the individual qualitative or quantitative component of the study. As a result, we could not easily determine the recruitment methods utilized by investigators and reported the lack of information in our findings (See Table 1). Another example was the very unbalanced descriptions of procedural and analytic methods between the quantitative and qualitative components of the study. Often investigators provided in-depth reports of the quantitative methods, analyses, and results, yet qualitative methods and analysis procedures were briefly described. In several cases, the amount of information provided about the qualitative component would not be sufficient for replication. O’Cathain et al[22] found this issue in their review of mixed methods studies in health services research and offered that it was indicative of the “historical dominance of quantitative methods in health services research.” One could argue that this is also true in the field of tobacco control research. Additionally, the lack of information impacts the ability to determine not only the quality of the qualitative and quantitative components, but the rigor of the overall study and the implications of the study findings. We recommend that authors using mixed methods approaches be consistent in their description of both the qualitative and quantitative strands of their study. They must ensure that they report the same information from each phase of the mixed methods design. Moreover, investigators can prioritize journals that recognize the richness of mixed methods designs and have less restrictive manuscript word limits.

### Lack of clear explanations of mixed methods procedures

The last issue is the lack of clear descriptions and explanations of mixed methods procedures. In the effort to assess the quality of mixed methods research, several investigators have created criteria to examine the rigor of the study.[16,17,22,76] O’Cathain et al[22] provided guidelines for Good Reporting of a Mixed Methods Study (GRAMMS). These quality criteria focus on how to clearly report seminal features of the mixed methods study design including the rationale, design, and the integration or “mixing” of the methods. The use of the major design names, explanation of the purpose or rationale of the design, as well as descriptions of the timing, priority, and integration were frequently omitted among this collection of studies hindering our ability to assess the quality of the research study. For instance, some studies’ mixing approaches involved using data from a previous study or embedding the current study within a larger parent study. Without an understanding of the how these qualitative and quantitative strands were integrated, we could not ascertain the synthesizability or quality of the research.[79] This is the central purpose of mixed methods research designs–the integration of both components.[18,80]Currently, mixed methods research experts refer to this issue as the “integration challenge.”[81] Specifically, although integration is required in mixed methods research to go beyond the separate quantitative and qualitative components; it is challenging to do so. We conducted this methodological review to assess studies that attempted to take on the integration challenge and thus articles that reported on only one component of their study did not meet our inclusion criteria. Moreover, investigators must fully appreciate that each mixed methods design is best suited for specific purposes/rationales. Thus, to successfully respond to the study’s purpose and research questions, one must logically and consistently implement and describe the various aspects of the study thoughtfully. Bryman[80] suggests that if investigators self-reflect on the original purpose/rationale for conducting their work, they can then use this information to conduct an integrative analysis. In essence, can researchers demonstrate that their integrated findings have enhanced their understanding of the phenomena under study beyond that of a single component of the study?[80] We recommend that tobacco control investigators become familiar with and master reporting guidelines such as GRAMMS[22] to meet the field of mixed methods research reporting and quality criteria and to effectively describe the rigor of mixed methods designed studies. The ability to articulate the important features of mixed methods research is paramount to advancing its use in tobacco control. Finally, given the word limits of journal articles, researchers should write robust descriptions of their study methods and restrict their embellishment of the introduction or background sections of their manuscripts.

It is important to note that the aforementioned issues are impacted by word and space limits set by journals and its respective publisher. These are critically important parameters that have been reported as an important barrier to publishing mixed methods research.[82] While there are tradeoffs for what should be highlighted in an article, guidelines exist for effectively reporting mixed methods research[22,23] to further advance the use of mixed methods designs in the field.

### Limitations

A few limitations of this methodological review must be considered in the interpretation of our results. Our work builds on established procedures for conducting rigorous methodological reviews within disciplinary contexts, including the journals selected for the review. To this end, these results represent articles published in five select journals; not all journals of the scientific tobacco control empirical literature and only those published in the English language. More importantly, our work contributes to understanding the current practices of tobacco control researchers with the use of the five major mixed methods designs identified by Creswell.[26,28] Articles that reported on only one component (quantitative or qualitative) of a study did not meet our definition of mixed methods research and was excluded from our sample. Additionally, the results represent a specific range in time during which our search was conducted. Although our methodological review covers a 12-year span (2004–2015), we did not include articles that have been published since January 2016.

## Conclusions

Mixed methods research designs can be of great promise in tobacco control research efforts. The central purpose of mixed methods designs is the integration of the quantitative and qualitative components. To this end, mixed methods research designs have the ability to provide findings of integrated data that are beyond the limitations of quantitative and qualitative data alone. Investigators utilizing mixed methods study designs can contribute to the complexities of youth and young adult behavior and prevention, the treatment of tobacco-caused problems such as dependence, including symptoms of withdrawal and cessation. The application of mixed methods designs can also provide the opportunity to discover new areas of research as the field grapples to address the emergence of a plethora of novel, non-conventional tobacco products specifically targeting youth and young adults such as the co-use or multiuse of tobacco products and other substances among young people. In the future, we recommend that tobacco control investigators be explicit about the study’s purpose and research design and use clear language in describing the many facets of their mixed methods approach. This would include the priority given to the qualitative or quantitative strand of the design as well the rationale for integrating or mixing the two strands. We believe our work is the beginning of a rich and fruitful discussion regarding the most meaningful use of mixed methods study designs when working with young people. Ultimately, the field of tobacco control needs more innovative research methods including innovative interventions in order to achieve the insights necessary to understand and overcome the complex issues of tobacco use among the world’s youth. Mixed methods research gives tobacco control researchers a powerful methodological strategy for taking on this challenge.

## Supporting information

S1 File Study dataset. (XLSX)

S2 File PRISMA 2009 checklist. (DOC)
